# Supplementary material for: Collective magnetism in an artificial 2D XY spin system
Source: Nat Commun. 2018 Jul 20;9:2850. doi: 10.1038/s41467-018-05216-2 (PMC6054668; doi:10.1038/s41467-018-05216-2)
Supplement: Supplementary file 1 — Supplementary Information [file 41467_2018_5216_MOESM1_ESM.pdf]

Collective magnetism in an artificial 2D XY spin system

Naëmi Leo[1,2,*], Stefan Holenstein[3,4], Dominik Schildknecht[1,2,5], Oles Sendetskyi[1,2], Hubertus Luetkens[3], Peter M. Derlet[5], Valerio Scagnoli[1,2], Diane Lançon[1,2,6], José R. L. Mardegan[7], Thomas Prokscha[3], Andreas Suter[3], Zaher Salman[3], Stephen Lee[8], & Laura J. Heyderman[1,2]

[1]Laboratory for Mesoscopic Systems, Department of Materials, ETH Zurich, 8093 Zurich, Switzerland.
[2]Laboratory for Multiscale Materials Experiments, Paul Scherrer Institut, 5232 Villigen PSI, Switzerland.
[3]Laboratory for Muon Spin Spectroscopy, Paul Scherrer Institut, 5232 Villigen PSI, Switzerland.
[4]Physik-Institut der Universität Zürich, Winterthurerstrasse 190, 8057 Zurich, Switzerland.
[5]Condensed Matter Theory Group, Paul Scherrer Institut, 5232 Villigen PSI, Switzerland.
[6]Laboratory for Neutron Scattering and Imaging, Paul Scherrer Institut, 5232 Villigen PSI, Switzerland
[7]Swiss Light Source, Paul Scherrer Institut, 5232 Villigen, Switzerland
[8]School of Physics and Astronomy, SUPA, University of St. Andrews, St. Andrews KY16 9SS, UK.

May 29, 2018

Supplementary Note 1

In this supplementary material, we provide a mean-field description of the μSR depolarisation signal for the antiferromagnetic stripe-ordered state of artificial dXY spin systems.

The applicability of a mean-field approach is discussed and the mean-field order parameter determined. Finally, the muon-spin precession and depolarisation is computed for a muon ensemble (distributed in a gold stopping layer as in the experiment) that probes the mean field of the emergent long-range-ordered stripe phase.

In the following, each macrospin is assumed to generate stray fields equivalent to those from a point dipole placed at the centre of the nanomagnet, with the magnetic moment given by $M = |\mathbf{M}| = VM_S$, where V is the volume of the nanoscale discs and M_S is the bulk magnetisation of thin-film permalloy.

Applicability of the Mean-Field Approach. The approximation of a static mean-field order parameter as a source of the muon-spin depolarisation disregards superparamagnetic and correlated fluctuations. Calculating the muon-spin depolarisation in an averaged mean field is justified only if the fluctuation rates of the macrospins ω_{dot} are much faster than the muon-spin precession frequency ω_μ. In this case, the muon spin does not follow the fast-fluctuating field, and therefore the magnetic field at the muon site can be approximated by its time-averaged value.

To determine the applicability of the mean-field approach, we establish when ω_μ is much smaller than ω_{dot} for the dXY spin systems: The precession frequency ω_μ of a muon at a height z above the macrospins is determined by the magnetic stray field at position $\mathbf{x} = (x, y, z)$ and, in the point-dipole approximation[1], this can be estimated using $\omega_\mu = \gamma_\mu \mathbf{B}_\mu \propto \gamma_\mu |\mathbf{M}|/z^3$, with γ_μ being the gyromagnetic ratio of the muon $\gamma_\mu/(2\pi) = 135.54\,\text{MHz T}^{-1}$. With the assumption that the macrospins are superparamagnetic, i.e. the interactions are strong enough to ensure $T_\text{C} > T_\text{B}$, the fluctuation frequency of the macrospins is proportional to the dipolar interaction, $\omega_{\text{dot}} \propto \gamma_{\text{Py}} |\mathbf{M}|/a^3$, where a is the lattice parameter and $\gamma_{Py}/(2\pi) = 29.5\,\text{GHz T}^{-1}$ is the gyromagnetic ratio of permalloy[2]. Considering

$$\omega_\mu \ll \omega_{\text{dot}} \qquad \Rightarrow \gamma_\mu \frac{|\mathbf{M}|}{z^3} \ll \gamma_{\text{Py}} \frac{|\mathbf{M}|}{a^3} \qquad \Rightarrow \left(\frac{a}{z}\right)^3 \ll \frac{\gamma_{\text{Py}}}{\gamma_\mu} \approx 200\,. \tag{1}$$

In our experiment, the lattice parameter a is given by the design of the artificial spin system and z denotes the height of the muons above the nanomagnets in the 80 nm-thick gold stopping layer. The muon stopping distribution $p_\mu(z)$ in the gold layer can be calculated using the program TRIM.SP (Ref. 3) .

Considering $(a/z)^3 < 40 = \frac{1}{5} \cdot \frac{\gamma_{\text{Py}}}{\gamma_\mu}$ as the criterion for the fast-fluctuation limit, and integrating the stopping probabilities $p_\mu(z)$ over z values between 0 nm and 80 nm that fulfil this criterion,

we obtain 60% for $a = 70\,\text{nm}$ (Sample Set 2). The same criterion yields 71% for $a = 55\,\text{nm}$ (Sample Set 3) and 40% for $a = 100\,\text{nm}$ (Sample Set 1). For muons that fulfil this criterion, the effective muon-spin depolarisation can be described by a time-averaged mean magnetic field caused by the antiferromagnetic correlations.

To quantitatively model the muon depolarisation in the time-averaged field caused by the emerging stripe order, we first establish the thermal average of the order parameter, and then track the time-dependent precession of the muon spin in this mean field.

Determination of the Effective Mean Field. We assume that each permalloy nanomagnet is described by a macrospin (point dipole) $\mathbf{S}_i$ confined to the xy plane. The Hamiltonian describing a dipolar-coupled spin system is:

$$H = D \sum_{i \neq j} \frac{1}{r_{ij}^3} \left[\mathbf{S}_i \cdot \mathbf{S}_j - 3(\mathbf{S}_i \cdot \hat{\mathbf{r}}_{ij})(\mathbf{S}_j \cdot \hat{\mathbf{r}}_{ij}) \right] , \tag{2}$$

with $r_{ij} = \mathbf{r}_i - \mathbf{r}_j$ and $\hat{\mathbf{r}}_{ij} = (\mathbf{r}_i - \mathbf{r}_j)/r_{ij}$. In the mean-field approximation, the single moments are rewritten as $\mathbf{S} = \langle \mathbf{S}_j \rangle + \delta \mathbf{S}_j$, where $\langle \mathbf{S}_j \rangle$ denotes the thermal spin average and $\delta \mathbf{S}_j$ represents the thermal fluctuations. Neglecting quadratic terms in δS_j, the resulting mean-field Hamiltonian for the dXY spin system in terms of the spin $\mathbf{S}_i$ and the thermal average $\langle \mathbf{S}_j \rangle$ is:

$$H_{\text{MF}} = 2D \sum_{i \neq j} \frac{1}{r_{ij}^3} \left[\mathbf{S}_i \cdot \langle \mathbf{S}_j \rangle - 3(\mathbf{S}_i \cdot \hat{\mathbf{r}}_{ij})(\langle \mathbf{S}_j \rangle \cdot \hat{\mathbf{r}}_{ij}) \right] . \tag{3}$$

The magnetic unit cell of the dXY model on a square lattice is given by a two-by-two spin plaquette[4] containing four spins. Therefore $\langle S_j \rangle$ has four components, and the Hamiltonian can be rewritten as

$$H_{\text{MF}} = \sum_{\text{Plaquettes}} \sum_{ml} \sum_{\mu,\nu} S_m^{\mu} M_{\mu\nu}^{ml} \langle S_l^{\nu} \rangle , \tag{4}$$

where $\mu, \nu = x, y$ and $l, m \in \{1, 2, 3, 4\}$ denote local indices enumerating the spins of a plaquette. In the mean-field approach the plaquettes do not interact, and thus the summation over the plaquettes in Eq. (4) is trivial. Using the Hamiltonian in Eq. (4), the partition function of a single plaquette can be derived which contains the inverse temperature $\beta = (k_{\text{B}} T)^{-1}$ and modified Bessel functions of the first kind $I_0(x)$:

$$K_i^{\nu} = \sum_{\nu j} M_{ij}^{\mu\nu} \langle S_j^{\nu} \rangle$$

$$Z(\beta) = \prod_{i=1}^{4} 2\pi I_0 \left(\beta \sqrt{(K_i^x)^2 + (K_i^y)^2} \right) \tag{5}$$

This leads to the following self-consistent equation Eq. (6) for the temperature-dependent spin components of the four-component mean-field order parameter $\langle S_p^\mu \rangle$, which was solved numerically:

$$\langle S_p^\mu \rangle = -\frac{\sum_{l\nu} M_{pl}^{\mu\nu} \langle S_l^\nu \rangle}{\sqrt{(\sum_{l\nu} M_{pl}^{x\nu} \langle S_l^\nu \rangle)^2 + (\sum_{l\nu} M_{pl}^{y\nu} \langle S_l^\nu \rangle)^2}} \frac{I_1 \left(\beta \sqrt{(\sum_{l\nu} M_{pl}^{x\nu} \langle S_l^\nu \rangle)^2 + (\sum_{l\nu} M_{pl}^{y\nu} \langle S_l^\nu \rangle)^2} \right)}{I_0 \left(\beta \sqrt{(\sum_{l\nu} M_{pl}^{x\nu} \langle S_l^\nu \rangle)^2 + (\sum_{l\nu} M_{pl}^{y\nu} \langle S_l^\nu \rangle)^2} \right)} \tag{6}$$

Here, the mean-field order parameter $\langle S_p^\mu \rangle$ of the dXY model on the square lattice exhibits an approximate square-root temperature dependence.

Muon-Spin Depolarization at a Fixed Point. A muon at position $\mathbf{x}$ with initial moment along x will precess in a static mean field according to

$$P(t, \mathbf{x}, T) = \cos^2(\theta(\mathbf{x})) + \sin^2(\theta(\mathbf{x})) \cdot \cos(\gamma_\mu |\mathbf{B}(\mathbf{x}, T)| t) \ , \tag{7}$$

where $\theta(\mathbf{x})$ denotes the angle between the magnetic field $\mathbf{B}(\mathbf{x}, T)$ and the initial direction x of the muon spin[5], i.e. $\theta(\mathbf{x}) = \arccos(\frac{B(\mathbf{x})_x}{B(\mathbf{x})})$. The stray field $\mathbf{B}(\mathbf{x}, T)$ can be factorised into different contributions $\mathbf{B}(\mathbf{x}, T) = \mu_0 M \cdot f(T) \cdot \mathbf{g}(\mathbf{x})$, separating the magnetic pre-factor $\mu_0 M$ (using SQUID magnetometry a value of 0.44 T was determined for discs with 40 nm diameter, i.e. Sample Set 2) and spatial dependence $\mathbf{g}(\mathbf{x})$ of the dipolar stray field, as well as the temperature dependence $f(T) \equiv \langle S_p^\mu \rangle$ of the mean-field order parameter from Eq. (6). With this factorisation, the relative angle $\theta(\mathbf{x})$ in Eq. (7) is a function of $\mathbf{g}(\mathbf{x})$ only. Therefore, the time-dependent depolarisation $P(t, \mathbf{x})$ needs to be calculated for one temperature $T < T_\mathrm{C}$ only. Results for any other temperature or magnetisation can be obtained by re-scaling with the pre-factor $f(T)$ or $\mu_0 M$, respectively. For Sample Sets 1 and 3 the value $\mu_0 M$ was estimated from the experimentally-determined values of T_C measured by muon-spin relaxation, using the relationship in Eq. (1) of the main text and the values of M and T_C of Sample Set 2.

Spatial Averaging of Muon-Spin Depolarisation. To estimate the muon-spin depolarization $P(t)$ measured in the experiment, $P(t, \mathbf{x})$ is averaged over all possible muon locations $\mathbf{x}$. Both the lateral spread in x and y, as well as the implantation profile along z need to be considered. Regarding the lateral spread in the xy plane, the muon-beam diameter (about 1.7 cm) is orders of magnitude larger than the lattice parameter (55 nm to 100 nm) of the experimentally investigated artificial dXY systems. Therefore, we can safely approximate the randomly-distributed lateral stopping positions of the muons by a uniform distribution of positions in the two-by-two spin magnetic unit cell. For the vertical averaging over possible stopping positions of the muons, i.e. z, we used the implantation distribution simulated with TRIM.SP as the weight.

Results and Discussion. Due to the spatial averaging of muons stopping at random positions x, y, z, the depolarisation function $P(t, T)$ for a mean-field stripe order does not show oscillatory behaviour but only decays with time, as the broad field distribution leads to a dephasing of the

signal. The damping contains (at least) two time scales, and can be fitted with a two-exponential decay function, i.e.

$$P(t) = g_0 + g_1 e^{-\lambda_1 t} + g_2 e^{-\lambda_2 t}\,, \tag{8}$$

with fractions g_i (with $i = 0, 1, 2$) and depolarisation rates λ_i (with $i = 1, 2$). The fit is performed leaving all parameters free.

From the fit, a slow dominant contribution $g_1 e^{\lambda_1 t}$ to the depolarisation function is obtained, and the fraction and relaxation rate for the second contribution are $g_2 \approx 0.5 g_1$ and $\lambda_2 \approx 6\lambda_1$, respectively. The temperature dependence of both contributions is the same, following the square-root increase of the mean-field stripe-order parameter below T_{C}, and the depolarisation rates are zero above T_{C} [Eq. (6)]. As shown in Figs. 3b,d,f in the main text, the determined magnitude of $\lambda_1(T)$ (blue solid line) compares well with the experimentally measured value of $\lambda_{\text{slow}}(T)$ (blue dots), which, at low temperatures, is of the order of $0.1\ \mu\text{s}^{-1}$ for the smaller nanodiscs ($d \approx 40\,\text{nm}$) and onnly deviates by a factor of two for the larger nanodiscs ($d = 70\,\text{nm}$). The calculated square-root-like temperature dependence of $\lambda_1(T)$, however, matches the experimental temperature dependence of $\lambda_{\text{slow}}(T)$ only roughly.

With the qualitative agreement between the experimental data and the muon-spin depolarisation described by a mean-field order parameter of dipolar-coupled XY moments on a square lattice, we therefore can relate $\lambda_{\text{slow}}(T)$ to the emergence of a static order parameter in a thermally-active artificial dXY spin system. In contrast, our mean-field model does not explain the contribution resulting in the $\lambda_{\text{fast}}(T)$-peak around T_{C} seen in the experiments, and therefore $\lambda_{\text{fast}}(T)$ must originate from fluctuation correlations or other effects that go beyond the mean-field description. Nevertheless, the mean-field description provides a connection between the slow contribution to the muon-spin relaxation and the emergent long-range order in our artificial dXY spin systems.

Supplementary References

1. Tsymbal, E. Evaluation of the magnetic dipolar fields from layered systems on atomic scale, *J. Magn. Magn. Mater.*, **130**, L6–L12 (1994). URL `http://dx.doi.org/10.1016/0304-8853(94)90651-3`.

2. Shaw, J., Nembach, H., Silva, T. & Boone, C., Precise determination of the spectroscopic g-factor by use of broadband ferromagnetic resonance spectroscopy, *Journal of Applied Physics* **114**, 243906 (2013). URL `https://doi.org/10.1063/1.4852415`.

3. Ziegler, J., *TRIM - Transport of Ions in Matter*, URL `http://www.srim.org/` (2013).

4. Prakash, S. & Henley, C. L., Ordering due to disorder in dipolar magnets on two-dimensional lattices, *Phys. Rev. B* **42**, 6574–6589 (1990). URL `http://dx.doi.org/10.1103/PhysRevB.42.6574`.

5. Yaouanc, A. & de Reotir, P., *Muon Spin Rotation, Relaxation and Resonance* (Oxford University Press, 2011).
